# Extracellular Vesicles Secreted by Mesenchymal Stromal Cells Exert Opposite Effects to Their Cells of Origin in Murine Sodium Dextran Sulfate-Induced Colitis

**DOI:** 10.3389/fimmu.2021.627605

**Published:** 2021-04-13

**Authors:** Anna Maria Tolomeo, Ignazio Castagliuolo, Martina Piccoli, Michele Grassi, Fabio Magarotto, Giada De Lazzari, Ricardo Malvicini, Federico Caicci, Chiara Franzin, Melania Scarpa, Veronica Macchi, Raffaele De Caro, Imerio Angriman, Antonella Viola, Andrea Porzionato, Michela Pozzobon, Maurizio Muraca

**Affiliations:** ^1^Department of Women’s and Children’s Health, University of Padova, Padua, Italy; ^2^Laboratory of Extracellular Vesicles as Therapeutic Tools, Fondazione Istituto di Ricerca Pediatrica Città della Speranza, Padua, Italy; ^3^L.i.f.e.L.a.b. Program, Consorzio per la Ricerca Sanitaria (CORIS), Padua, Italy; ^4^Department of Molecular Medicine, University of Padova, Padua, Italy; ^5^Laboratory of Tissue Engineering, Fondazione Istituto di Ricerca Pediatrica Città della Speranza, Padua, Italy; ^6^Laboratory of Stem Cells and Regenerative Medicine, Fondazione Istituto di Ricerca Pediatrica Città della Speranza, Padua, Italy; ^7^Instituto de medicina traslacional, trasplante y bioingenieria (IMeTTyB-CONICET), Buenos Aires, Argentina; ^8^Department of Biology, University of Padova, Padua, Italy; ^9^Laboratory of Advanced Translational Research, Veneto Institute of Oncology IOV–IRCCS, Padua, Italy; ^10^Department of Neurosciences, University of Padova, Padua, Italy; ^11^Department of Surgery, Oncology and Gastroenterology, University of Padova, Padua, Italy; ^12^Department of Biomedical Sciences, University of Padova, Padua, Italy

**Keywords:** inflammatory bowel disease, mesenchymal stromal cells, extracellular vesicles, macrophage polarization, sodium dextran sulfate, immunomodulation

## Abstract

Several reports have described a beneficial effect of Mesenchymal Stromal Cells (MSCs) and of their secreted extracellular vesicles (EVs) in mice with experimental colitis. However, the effects of the two treatments have not been thoroughly compared in this model. Here, we compared the effects of MSCs and of MSC-EV administration in mice with colitis induced by dextran sulfate sodium (DSS). Since cytokine conditioning was reported to enhance the immune modulatory activity of MSCs, the cells were kept either under standard culture conditions (naïve, nMSCs) or primed with a cocktail of pro-inflammatory cytokines, including IL1β, IL6 and TNFα (induced, iMSCs). In our experimental conditions, nMSCs and iMSCs administration resulted in both clinical and histological worsening and was associated with pro-inflammatory polarization of intestinal macrophages. However, mice treated with iEVs showed clinico-pathological improvement, decreased intestinal fibrosis and angiogenesis and a striking increase in intestinal expression of Mucin 5ac, suggesting improved epithelial function. Moreover, treatment with iEVs resulted in the polarization of intestinal macrophages towards and anti-inflammatory phenotype and in an increased Treg/Teff ratio at the level of the intestinal lymph node. Collectively, these data confirm that MSCs can behave either as anti- or as pro-inflammatory agents depending on the host environment. In contrast, EVs showed a beneficial effect, suggesting a more predictable behavior, a safer therapeutic profile and a higher therapeutic efficacy with respect to their cells of origin.

## Introduction

Inflammatory Bowel Diseases (IBD) such as Ulcerative Colitis and Crohn’s Disease are chronic inflammatory diseases of the gastro-intestinal tract. Although the origin of IBD remains obscure, the widely believed hypothesis is that environmental factors or infections can alter the barrier function of the epithelium, leading to loss of immune tolerance to intestinal antigens (1, 2). The immune response in the intestine is a tightly controlled balance between innate and adaptive effector responses and negative regulatory pathways. Dysregulation of the immune response and alteration of the equilibrium between protective immunity and tolerance to self-antigens and commensal bacteria have been emphasized as pathogenic factors (1, 2). Drugs used to suppress the immune response and reduce tissue inflammation are not always effective and are often associated with more or less serious side effects (3). Mesenchymal stromal cells (MSCs) have shown beneficial effects in animal models of IBD and have been approved by the European Medicines Agency for the treatment of fistulas in Crohn’s disease following a successful phase II/III clinical trial (4). In their clinical application for the treatment of immune disorders, however, administrated MSCs might encounter a biased cytokine milieu *in vivo*, which could actually render MSCs immune-enhancing (5). Indeed, in some conditions MSCs could promote the proliferation of suboptimally activated T cells (6, 7).Therefore, MSCs appear to respond to environmental signals possibly resulting in unpredictable opposite behavior *in vivo*.

It is now well established that several beneficial effects of MSC transplantation are mediated by paracrine signaling (8–10), and that such signals are mostly convoyed *via* membrane vesicles released by the cells, named extracellular vesicles (EVs) (11–13). EVs are a heterogeneous population of nanoparticles up to 1 μm in size, including exosomes and microvesicles, carrying both effector molecules and RNAs (14). The discovery that EVs secreted by MSCs can reproduce some immunomodulatory and pro-regenerative effects of their cells of origin has prompted investigations on the use of these cell products as therapeutic tools, and indeed their beneficial effects have been confirmed in several animal models of organ and tissue injury (15). In particular they were effective in improving both clinical and histological signs of colitis in animal models (15). We also observed similar therapeutic effects of MSCs and EVs in hyperoxia-induced lung injury (16).

Following the recognition of the therapeutic potential of exosomes, several researchers are trying to modify their composition to enhance specific biological effects on target cells. Several authors have shown that the immunomodulatory activity of MSCs is affected by cytokine priming, both *in vitro* and *in vivo* (17–20). However, only few studies have investigated the effects of MSC priming by pro-inflammatory cytokines on the biological activity of released EVs (21, 22). Here, we investigated the effects of both MSCs and EVs, with and without priming with pro-inflammatory cytokines, in a well-established murine model of IBD. Surprisingly, we observed a divergent effect of MSC-EVs vs. their parent cells, a difference that was amplified by cytokine conditioning.

## Materials and Methods

### Murine MSC Isolation and Culture

Murine MSCs were isolated by flushing the bone marrow of C57BL6/J mice. Cells were cultured in 25 cm^2^ tissue culture flasks at a concentration of 2.00E+6 cells/cm^2^. MSCs were fed in Dulbecco’s modified eagle medium low glucose [1g/L] (DMEM; Gibco by Life Technologies) enriched with 10% fetal bovine serum (FBS; Gibco by Life Technologies), [100 U/mL] penicillin and streptomycin (P/S; Gibco by Life Technologies), [100 mg/mL] L-glutamine (Gibco by Life Technologies) and incubated at 37°C, 5% CO2. After 48 hours, the non-adherent cells were removed. After reaching 80% confluence, the adherent cells were trypsinized (0.05% trypsin at 37°C for 3 minutes), harvested and expanded in larger flasks. MSC at passage 5 were screened by flow cytometry (LSRFortessa (BDBiosciences) for the expression of SCA-1, CD31 and CD34 (BD Pharmingen, Oxford, UK) and used to perform the experiments. MSCs were grown up till reach 80% of confluency in ventilated cap flask. Growth medium was substituted with DMEM low glucose [1g/L] supplemented with 10% FBS, 2mM glutamine, 100 U/ml penicillin/streptomycin, without (for naïve MSCs) or with a pro-inflammatory cytokine cocktail (for induced MSCs: iMSC) composed by IL-6 [20 ng/mL], TNF-α [25 ng/mL] and IL-1β [25 ng/mL] (Peptrotech) for 24 hours. This medium was changed with DMEM low glucose supplemented with 2mM glutamine, 100 U/ml penicillin/streptomycin for 24 hours before collection for EV isolation (20, 23).

### Extracellular Vesicles by Ultrafiltration

Culture medium (CM) was centrifuged at 1200 rpm for 6 minutes to discard dead cells and debris and filtered through 0.22 μm filter (filter unit syringe driven, Millex-GP). Supernatant was loaded onto an Amicon filter device (Amicon filters Ultra-15, regenerate cellulose 100,000 NMWL; Merck Millipore). The filter unit was centrifuged at 3200g at 4°C for 15 minutes and the concentrate was collected.

### Measurement of Extracellular Vesicles Concentration and Size Distribution

Particle concentration and size distribution were analyzed by Tunable Resistive Pulse Sensing (TRPS) technology with the qNano instrument (Izon Science, Christchurch, New Zealand). In this system, a membrane including a tunable submicron-sized pore separates two fluid chambers, one containing the sample to be analyzed, the other an electrolyte solution. A voltage is applied across a membrane, resulting in an ionic current. While passing through the pore, the particles generate a “blockade” event, which in turn generates a pulse that is directly proportional to the particles’ volume, while the blockade rate is related to particle concentration. In the present setup, a NP150 membrane was used. The concentration of particles was standardized using a CPC100 calibration solution diluted 1:10000 (110 nm mean carboxylate polystyrene beads; raw concentration 1.00E+12).

### Functional Assays

#### Macrophage Functional Assay

RAW 264.7 murine macrophages (passages 12-15) were cultured in DMEM High Glucose 5% FBS. For the assay, cells were seeded in sextuplicate in a 96-well plate (40.000cel/well) for 24h and were stimulated with or without LPS from E. coli O111:B4 (L4391, Sigma) at a dose of 10ng/mL, plus dexamethasone (D4902, Sigma) at a dose of 1µg/mL or plus nEV or iEV (5,00E+7, 5,00E+8, 5,00E+9 EV) for 16h. After that, cell culture supernatant was taken and analyzed for IL-10 concentration following manufacturer’s instructions (mouse IL-10 ELISA kit DY417, R&D).

#### Endothelial Functional Assay

80 µl of Matrigel Matrix (Corning) were seeded in the 96-well plate and left to polymerize at 37°C, 5% CO2 for at least 30 minutes. SVEC4-10 cells with 5,00E+9 EVs were resuspended in 96µl DMEM, supplemented with 10% FBS seeded on the solidified matrix and incubated for 4 hours at 37°C 10% CO2. DMEM low glucose with 10% heat-inactivated FBS was used as control. After the incubation, pictures were taken with a phase contrast inverted microscope (Olympus) and analysis were performed with ImageJ Angiogenesis Analyzer.

### Western Blot Analysis

For the western blot analysis, both naïve and induced mMSC and EV were lysed in RIPA buffer and then incubated with Laemmli buffer with β-mercaptoethanol for 5 min at 95°C, for complete protein denaturation. Then, samples were loaded and resolved in an SDS-polyacrylamide 4-12% gel at 140V and blotted using semi-dry transfer for 7 min at 25V to polyvinylidene difluoride membranes (PVDF) (GE Healthcare Life science). Membranes were blocked with 5% BSA in TBS-Tween for 1h at room temperature and then incubated with primary antibodies overnight: mouse monoclonal ALIX antibody 1:500 (MA183977, Thermofisher), rabbit polyclonal TSG-101 antibody 1:1000 (ab-125011, Abcam), diluted in 1% BSA in TBS-Tween. After washing, membranes were incubated with secondary antibodies: goat- anti rabbit-HRP 1:5000 (65-6120, Thermofisher) and goat anti-mouse-HRP 1:5000 (62-6520, Thermofisher) for 1h at room temperature. After washing, bands were evidenced by means of ECL Plus Western blotting analysis system (32134, Thermofisher).

### Transmission Electron Microscopy (TEM)

One drop of EVs solution (about 25µl) was placed on 400 mesh holey film grid; after staining with 1% uranyl acetate (for 2 minutes) the sample was observed with a Tecnai G2 (FEI) transmission electron microscope operating at 100 kV. Images were captured with a Veleta (Olympus Soft Imaging System) digital camera.

### Mouse Model of Colitis

C57BL/6J mice were purchased from Charles River Laboratories (Calco, Italy). All mice used as primary cell donors or recipients were between 8 and 12 weeks of age. Procedures involving animals and their care conformed to institutional guidelines in compliance with national (4D.L. N.116, G.U., suppl. 40, 18-2-1992) and international (EEC Council Directive 2010/63/UE; National Institutes of Health Guide for the Care and Use of Laboratory Animals) law and policies. The protocol was approved by the Italian Ministry of Health, Division of Veterinary Medicine (protocol n°861/2016-pr, risp. a prot c35de.2 #195042387#). All efforts were made to minimize the number of animals used and their suffering. Colitis was induced in C57BL/6N mice by administration of 3% dextran sulfate sodium (DSS) (molecular mass, 40 kDa; Sigma Aldrich) in drinking water for 6 days followed by 3 days on plain water. Mice with colitis were divided into five treatment groups as described in **Table 1** and received an intraperitoneal injection (IP) of MSCs on days 4 and 8 or of EVs on days 4, 6 and 8. Both MSCs and EVs were suspended in 200 μL of PBS. Control mice received PBS only. To administer an amount of EVs approximately proportional to the number of injected MSCs, we calculated the EV dose based on the following assumptions: i) the transplanted MSCs remain active in the host tissue for about 24-48 hours (24, 25); ii) the transplanted MSCs produce an amount of EVs comparable to that produced *in vitro* in standard 2D culture conditions in this time period (about 1.00E+3 EVs per cell according to our experience). The severity of colitis was assessed daily by measurement of weight loss and of disease activity index (DAI). DAI was calculated on the degree of diarrhea and of visible fecal blood as described by Cooper et al. (26). Mice were euthanized on day 10. The entire colon was removed, and colon length was measured. The colon was opened longitudinally and rinsed with physiological saline to remove fecal residues. Tissue samples were stored in liquid nitrogen for subsequent analysis or embedded in paraffin.

**Table 1:** Experimental groups.

Group	N	Intraperitoneal (IP) Treatment
Healthy, untreated (UT)	5	none
Colitis + vehicle only (PBS)	5	200μL/administration
Colitis + MSCs	5	4.00E+6/administration
Colitis + iMSCs	5	4.00E+6/administration
Colitis + nEVs	5	1.00E+9/administration
Colitis + iEVs	5	1.00E+9/administration

### Histopathological Analysis

The colon, from the rectum to the ileo-cecal junction, was entirely sampled for histopathological evaluation. It was cut longitudinally and fixed in 10% buffered formalin for 2 days. The colon was then paraffin-embedded; 5-µm-thick sections were sliced and stained with Hematoxylin and eosin. The colonic mucosa had a quite patchy aspect, showing the alternation of lesions and almost normal mucosa. The intestinal lesions were evaluated through the following score: 0, no inflammation or crypt damage; 1, mild inflammation and damage of the basal 1/3 of crypts; 2, moderate inflammation and damage of the basal 2/3 of crypts; 3, severe inflammation with total crypt loss, although in the presence of surface epithelium; 4, severe inflammation with total crypt loss, in the absence of surface epithelium. The total colitis score was then calculated by multiplying the above score for the percentage of damaged colonic mucosa. In particular, this percentage was calculated by dividing the total length of the injured colonic areas for the total length of the colon (from the rectum to the ileo-cecal junction and without considering the cecum).

### Lymph Node Cell Isolation and Flow Cytometry

Single cells suspensions were prepared from mesenteric lymph nodes (MLN) by forcing the organs through an 80µm mesh (Sigma-Aldrich). Cells were washed twice with PBS and then suspended in Flow Cytometry Staining Buffer (eBioscience). To block unspecific binding of antibodies, cell suspensions were incubated with an anti‐CD16/32 mAb (2.4G2, eBioscience) for 15 min on ice and then stained with combinations of the following fluorochrome-labeled antibodies against surface markers for 30 min on ice: CD3 (17A2, eBioscience), CD4 (GK1.5, eBioscience), CD69 (H1.2F3, Abcam), and CD25 (PC61.5, eBioscience). Intracellular staining for FoxP3 (FJK-16s, eBioscience) was performed using the FoxP3/Transcription factor staining buffer set (eBioscience) according to the manufacturer’s instructions. Flow cytometric analysis was performed using a FACSCalibur based on CellQuest software (BD-Becton Dickinson, Franklin Lakes, USA). T cell effectors (Teff) were defined by: CD3, CD4, CD69+. T cell regulators (Treg) were defined by: CD3, CD4, CD25, FoxP3+.

### Immunohistochemistry

#### Slides Deparaffinization

Slides were incubated twice for 5’ in xylene, and then hydrated in a series of ethanol solution at decreasing concentration (100%/100%/95%/80%) for 3 minutes each and then placed in distilled water. To completely deparaffinize samples without disrupting their morphology and without damaging surface epitopes, slices were placed in a pre-heated bath at 90°C with a Trisodium Citrate pH 6,03 buffer solution for 30 minutes. Slides were rinsed with PBS-Tween20 0.05% for two minutes.

#### Immunofluorescence

A solution of glycine 0,1 M to block unspecific binding with antibodies, and then permeabilized with a solution of 1% BSA and 0.02% NP40 in PBS for 1h at room temperature. Primary antibodies were diluted in 1% BSA solution in PBS and used accordingly to **Table 2**. Slides were washed in PBS and incubated with secondary antibodies at 37° C for 1 hour. Hoechst (H3570 Life technologies) 1:10000 in PBS was used for 15 minutes to mark nuclei and mounting medium (Dako S3023) was used to seal the slides. Pictures were taken using confocal LSM 800 Zeiss at 20X of magnitude. Cells positive for each antibodies and Hoechst were counted and expressed as percentage of positive cells/field.

**Table 2:** Antibody list.

Antibodies	Dilution	Incubation condition	Clone	Ref
CD31	1:50	O.N. 4°C	JC70A	M0823 (Dako)
Rabbit anti-iNOS	1:80	O.N. 4°C	EPR16635	Ab178945 (abcam)
Rabbit anti-CD163	1:50	O.N. 4°C	EPR19518	Ab182422 (abcam)
Mouse anti-Muc5ac	1:80	O.N. 4°C	45M1	MA512178 (Invitrogen)
Rat anti-CD45	1:100	O.N. 4°C	IBL-3/16	MCA1388 (Biorad)
Goat anti-Mouse IgG Secondary Antibody, Alexa Fluor 594	1:200	1 h 37°C	Polyclonal	A11005 (Life Technologies)
Goat anti-Rat IgG Secondary Antibody, Alexa Fluor 488	1:200	1 h 37°C	Polyclonal	A11006 (Life Technologies)
Goat anti-Rabbit IgG Secondary Antibody, Alexa Fluor 594	1:200	1 h 37°C	Polyclonal	A21442 (Life Technologies)

#### Immunohistochemistry Was Performed With CD31

0.3% hydrogen peroxide for 10 min at room temperature was used to remove endogenous peroxidase activity and blocking serum for 30 min at room temperature. After primary antibody incubation with anti-mouse/rabbit serum (DAKO EnVision - TM Peroxidase, Rabbit, Dako Corporation, Carpinteria, CA) for 30 min at room temperature, 3,3=-diaminobenzidine (Sigma- Aldrich, Milan, Italy) was used. Counterstained with hematoxylin was performed. Negative controls were also done. Pictures were taken with a Leica DM4500B microscope coupled with a DFC320R2 camera for image acquisition and analyzed using Fiji software Color deconvolution/H DAB to analyze the signal of CD31. Threshold software was used to quantify area with positive CD31 signal, data were expressed as percentage of tissue covered per field.

#### Sirius Red

To quantify Collagen I on our samples, staining for Sirius red was performed. Hematoxylin staining for nuclei using Gills Hematoxylin solution nr.3 (Bio Optica 05-M06015) followed by staining with Sirius red solution (Sigma Cat #P6744-16A) following manufacturer’s instruction. Mounting medium (Fluka 03989) was used. Ten random pictures were taken at 10X magnitude with Leica B5000 inverted microscope and quantification of collagen was performed with Fiji software (plugin color deconvolution for Fast Red Fast Blu DAB), that was quantified using Fiji’s Threshold software. Threshold provide us the percentage of area stained in comparison with the dimension of the picture and results were expressed as Collagen I deposition/field (%).

### Quantitative Real Time pCR

RNA was extracted using Rneasy Kit (Qiagen) from samples previously snap frozen in liquid nitrogen. Quantification and assessment of RNA quality were obtained with Nanodrop 2000 (Thermoscientific). High Capacity cDNA Reverse Transcription kit (Applied Biosystems) was used and qRT-PCR was performed with Platinum^®^ Syber Green Mix (Lifetechnologies) using Roche thermocycler. A relative quantification (RQ) was calculated by ΔΔCt methods. Beta2microglobulin was used as reference gene for normalization. Primer sequences used are listed in **Table 3**. All graphs displayed were produced with GraphPad software 6. Data are displayed as means ± standard deviation

**Table 3:** Primer list.

Primers	Forward	Reverse	NM	Product length (bp)
*NOS2*	GCAGGTCTTTGACGCTCGGA	ATGGCCGACCTGATGTTGCC	NM_010927.3	105
*Arg1*	AGACCACAGTCTGGCAGTTGG	AGGTTGCCCATGCAGATTCCC	NM_007482.3	136
*CD31*	AGCCTCACCAAGAGAACGG	GTGGGGACAGGCTCATAAATAC	NM_001032378.2	150
*Ang1*	CAGTGGCTGCAAAAACTTG	AGACTGGTTCCTATCTCAAGC	NM_001286062.1	119
*Vegf*	CTCCACCATGCCAAGTGGTC	GTCCACCAGGGTCTCAATCG	NM_001025250.3	126
B2micro	GCTTCAGTCGTCAGCATGG	CAGTTCAGTATGTTCGGCTTCC	NM_009735.3	149

### Statistical Analysis

Normally distributed variables are presented as mean ± SD. Comparisons of categorical variables were carried out using one-way ANOVA followed by Bonferroni’s Multiple Comparison Test. P<0.05 was assumed statistically significant. Statistical analysis was performed by the use of IBM SPSS Statistics version 20(SPSS Inc., Chicago, IL, USA).

## Results

### Characterization of MSCs and EVs

**Figure 1:**
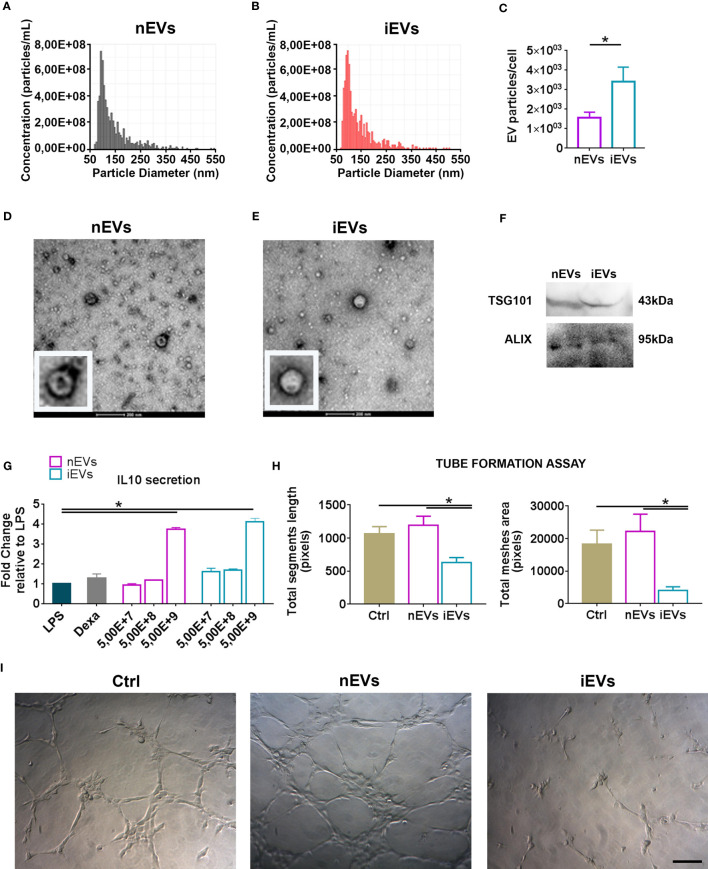
Characterization of extracellular vesicles derived from mesenchymal stromal cells. **(A)** Representative size distribution of nEV and of **(B)** iEV analyzed by Resistive Pulse Sensing. **(C)** Number of nEVs and of iEVs secreted per cell. Result are mean ± standard error (n = 5 independent experiments, *P < 0.05). **(D)** Transmission electron microscopy analysis of freshly nEVs **(E)** and of freshly iEVs; scale bar refers to 200 nm. **(F)** Western Blot of nEVs and iEVs for TSG101 and Alix. **(G)** Macrophage functional assay. IL-10 quantification after LPS cell treatment. (n = 3 independent experiments, *P < 0.05). **(H, I)** Endothelial functional assay. Segment length and meshes area were measured after nEVs and iEVs treatment. **(I)** Representative phase contrast images. Scale bar: 100μm.

The naïve murine MSCs (nMSCs) and the induced MSCs (iMSCs) fulfilled the minimal criteria that define MSCs (27); they appeared spindle shaped and distributed as swirling (**Supplementary Figures 1A–C**). Moreover, both nMSCs and iMSCs were positive for the classical mesenchymal markers Sca1-A, CD105, CD44, CD29 and negative for the hematopoietic markers CD45, CD117 as demonstrated by the flow cytometric analysis (**Supplementary Figures 1B, D**). EVs expressed CD9, CD63 (23) as well as ALIX and TSG101 (**Figure 1F**). Tunable Resistive Pulse Sensing (TRPS) analysis of EVs isolated from nMSCs and iMSCs, demonstrated two homogeneous populations with particle size mostly below 100 nm, with no significant differences between naïve EVs (nEVs) and induced EVs (iEVs) (**Figures 1A, B**). Transmission electron microscopy (TEM) identified EVs as a group of heterogeneous spheroids with the size ranging from 30 to 100 nm (**Figures 1D, E**). No apoptotic bodies were detected (28). Notably, EV particles/cell was increased by approximately three-fold in the medium of cytokine-conditioned MSCs (**Figure 1C**).

The immunomodulatory activity of EVs was evaluated *in vitro* by analyzing their dose-response effects on the production of the anti-inflammatory cytokine IL-10 by LPS-stimulated macrophages. Three different doses were tested (5,00E+7, 5,00E+8, 5,00E+9 nanoparticles respectively). Both nEVs and iEVs significantly increased IL-10 secretion after LPS treatment at the highest dose (**Figure 1G**). The effect of iEVs on angiogenesis *in vitro* was analyzed with a tube formation assay using the highest EV dose of the previous immunological test. The anti-angiogenic effect of iEVs is shown in **Figure 1H**: segment length and meshes area were drastically decreased in iEV treated cells. Representative pictures of the tube morphology derived from nEV and iEV treatment are shown in panel 1I.

### EVs but Not MSCs Administration Ameliorates Clinicopathological Signs of Colitis

**Figure 2:**
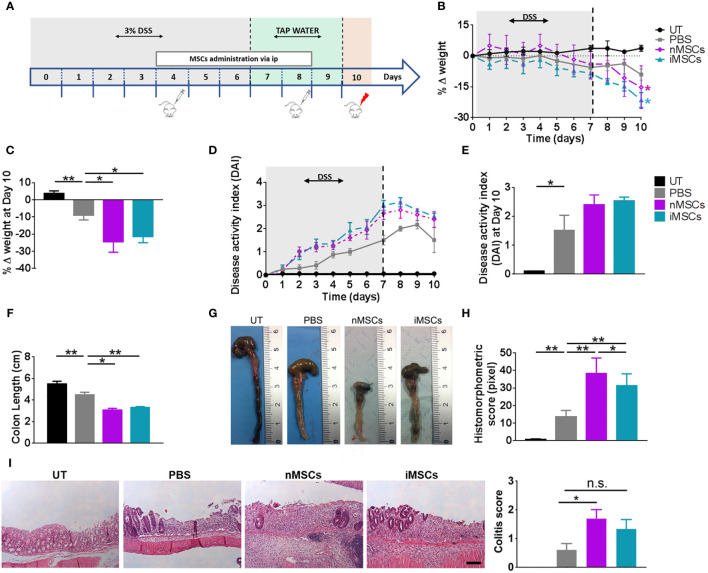
MSC administration worsens colitis in mice. **(A)** Experimental design. DSS was given in drinking water for 6 days followed by three days on plain water. MSCs were injected intraperitoneally on days 4 and 8. Weight loss **(B)**, Percent weight loss **(C)** and disease activity scores **(D)** were determined daily. **(E)** Disease activity index at 10 days. Colon length **(F, G)**, histomorphometric score **(H)** and colitis score **(I)** were determined on day 10. Scale bars, 50 μm. Results are mean ± standard error (n = 5). *P < 0.05, **P < 0.01, n.s. = not significant.

**Figure 3:**
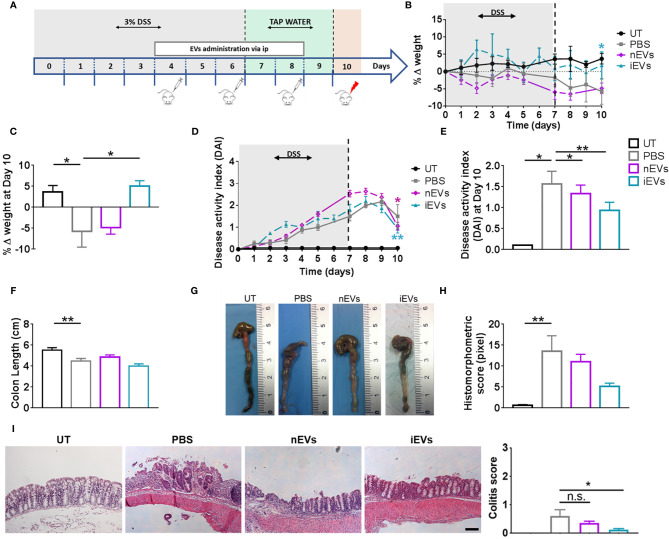
EVs improve the clinicopathological signs of colitis. **(A)** Experimental design. DSS was given in drinking water for 6 days followed by three days on plain water. EVs were injected intraperitoneally on days 4, 6 and 8. Weight loss **(B)**, Percent weight loss **(C)** and disease activity scores **(D)** were determined daily. **(E)** Disease activity index at 10 days. Colon length **(F, G)**, histomorphometric score **(H)** and colitis score **(I)** were determined on day 10. Scale bars, 50 μm. Results are mean ± standard error (n = 5). *P < 0.05, **P < 0.01, n.s. = not significant.

Treatment with DSS was administered in drinking water for 6 days. MSCs were injected at days 4 and 8 (**Figure 2A**) and EVs at days 4, 6 and 8 (**Figure 3A**). MSC dose was in the order of 10^6^, similar to the one shown to be effective in previous publications on the treatment of DSS-induced colitis (29–31). We observed that the administration of both nMSCs and iMSCs worsened the typical signs of colitis evidenced by weight loss and colon length reduction with a trend to increased DAI (**Figures 2B–E**). Macroscopic examination of colons showed strong hyperemia and inflammation associated with the decrease in colon length (**Figures 2F, G**). Histological analysis of colon mucosa showed patchy areas of inflammatory infiltrate with tissue necrosis and ulcer formation. In line with these results, the severity of histological injury was increased in MSC-treated groups (**Figures 2H, I**).

With regards to the treatment with EVs, we set the doses in our previous work (32). We found that nEVs did not ameliorate body weight loss and colon length reduction, although it resulted in a modest but significant improvement in DAI (**Figures 3B–E**). Histomorphometric analysis did not show any appreciable effect of nEVs on colon mucosa (**Figure 3I**). However, treatment with iEVs was associated with normalization of body weight and with a significant improvement in DAI, although with a non-significant change in colon length (**Figures 3F, G**). Moreover, the histomorphometric and colitis score analysis confirmed the beneficial effect of iEVs evidenced by a strong reduction of necrotic mucosal surface (**Figures 3H, I**).

### Different Effects of Unprimed or Cytokine-Primed MSCs and of Their Secreted EVs on Intestinal Macrophage Polarization

**Figure 4:**
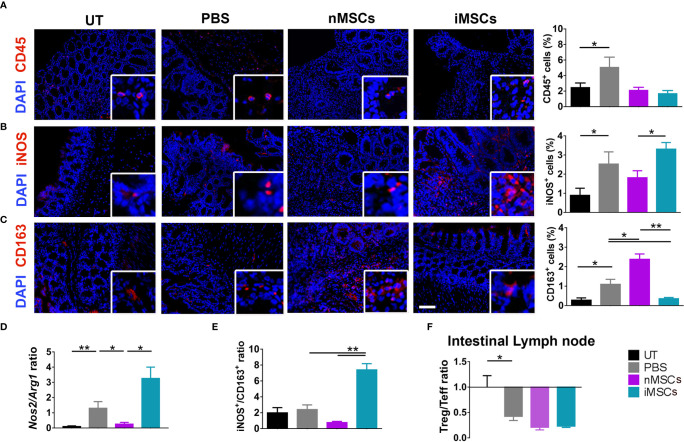
Administration of cytokine-conditioned MSCs is associated with pro-inflammatory polarization of intestinal macrophages. **(A)** CD45 pan lymphocyte IF. CD45 positive cells are equally present in nMSCs and iMSCs treated mice. **(B, C)** Immunofluorescence labeling of macrophage polarization markers in colon mucosa: IF (left), quantification (right). iMSCs induce iNOS protein, M1 marker. **(D)** qRT-PCR M1/M2 ratio: iEVs administration results in a significant presence of anti-inflammatory *Arg1* gene. **(E)** protein M1/M2 ratio. **(F)** Treg/Teff ratio in the intestinal lymph node. Scale bar: 50μm. Results are mean ± standard error (n = 5). *P < 0.05, **P < 0.01.

**Figure 5:**
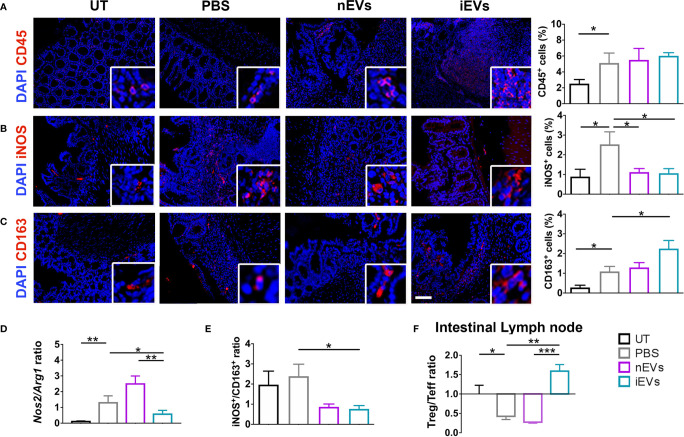
EV administration reverts macrophage polarization to an anti-inflammatory phenotype and improves M1/M2 and Treg/Teff ratio. **(A)** CD45 pan lymphocyte IF. CD45 positive cells are equally present in PBS, nEVs and iEVs treated mice. **(B, C)** Immunofluorescence labeling of macrophage polarization markers in colon mucosa: IF (left), quantification (right). iEVs induce CD163 protein, M2 marker. **(D)** qRT-PCR M1/M2 ratio: iEV administration results in a significant presence of anti-inflammatory *Arg1* gene. **(E)** protein M1/M2 ratio. **(F)** Cytofluorimetric analyses of freshly isolated cells form intestinal lymph node: Treg/Teff ratio. Scale bar: 50μm. Results are mean ± standard error (n = 5). *P < 0.05, **P < 0.01, ***P < 0.001.

Induction of colitis was associated with an increased infiltration of CD45^+^ cells in colon tissue. This parameter did not show significant differences between the different treatments (nMSC vs iMSC and nEVs vs iEVs) (**Figures 4A** and **5A**). Specifically, nMSCs administration tended to induce an anti-inflammatory polarization in macrophages. This effect was statistically significant when analyzing the Nos2/Arg1 ratio by qRT-PCR (**Figure 4D**) and there was a trend when analyzing iNOS^+^/CD163^+^ cell ratio by immunofluorescence (**Figure 4E**). Conversely, the administration of iMSCs polarized macrophages to a pro-inflammatory phenotype. Indeed, both the ratio of the genes *Nos2/Arg1* and of the proteins iNOS^+^/CD163^+^ cell ratio was significantly higher (**Figures 4B–E**). No effect of MSC administration (either naïve or induced) was observed on the Treg/Teff ratio at the level of the intestinal lymph node as the ratio were similar to that of the PBS group (**Figure 4F**). With regards to the administration of nEVs, they did not show a clear effect on macrophage polarization nor on Treg/Teff ratio, although a reduction of iNOS expression (M1 marker) was observed by immunofluorescence of colon tissue (**Figures 5B–F**). However, administration of iEVs promoted macrophage polarization to an anti-inflammatory phenotype, as shown by a decrease in both the *Nos2/Arg1* ratio and iNOS^+^/CD163^+^ cell ratio. In particular, immunofluorescence analysis showed a significant reduction of the M1 marker iNOS and a significant increase of the M2 marker CD163 in colon tissue of iEV-treated animals (**Figures 5B–E**). This finding was associated with a significant increase in the Treg/Teff ratio at the level of the intestinal lymph node (**Figure 5F**).

### Different Effects of MSCs and of Their Secreted EVs on Collagen Deposition and Angiogenesis

**Figure 6:**
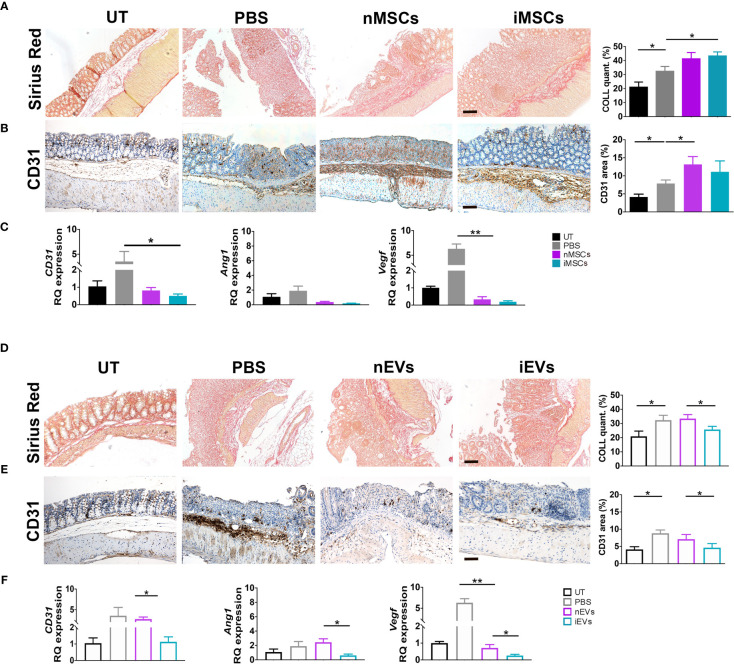
MSCs and iEVs exert opposite effect on fibrogenesis and angiogenesis in mice with colitis. **(A)** Collagen deposition in colon mucosa: Sirius red (left), quantification (right). **(B)** Quantitative CD31 staining as a measure of angiogenesis in colon mucosa: immunocytochemistry (left), quantification (right). **(C)** qRT-PCR of *CD31, Angiopoietin, Vegf* genes. All the genes were down regulated in nMSCs and iMSCs injected mice. **(D)** Collagen deposition in colon mucosa: Sirius red (left), quantification (right). **(E)** Quantitative CD31 staining as a measure of angiogenesis in colon mucosa: immunocytochemistry (left), quantification (right). **(F)** qRT-PCR of *CD31, Angiopoietin, Vegf* genes. With the exception of *CD31* in nEVs, *Angiopoietin* and *Vegf* were down regulated in nEVs and iEVs injected mice. Scale bar: 50μm. Results are mean ± standard error (n = 5). *P < 0.05, **P < 0.01.

Both nMSCs and iMSCs increased collagen deposition in colon mucosa, however statistical significance was achieved only with the administration of the latter (**Figure 6A**). The presence of the endothelial marker CD31 was used to assess angiogenesis by qRT-PCR and immunocytochemistry. CD31 expression was increased at protein level, although to a significant extent only in the group treated with unprimed MSCs (**Figure 6B**). Gene expression of CD31, *Ang1* and *Vegf* was down regulated in both naïve and primed MSCs groups (**Figure 6C**). Conversely, both collagen deposition and angiogenesis were significantly reduced in the iEV-treated group. In the same group of mice, CD31, *Ang1* and *Vegf* gene expression decreased as well as CD31 detected as protein in the colon tissue. nEVs administration had no effect on both parameters (**Figures 6D–F**). This finding suggests that iEVs are best suited as an anti-angiogenic agent.

### Effects of MSCs and of Their Secreted EVs on Mucin 5ac in Colon Mucosa

**Figure 7:**
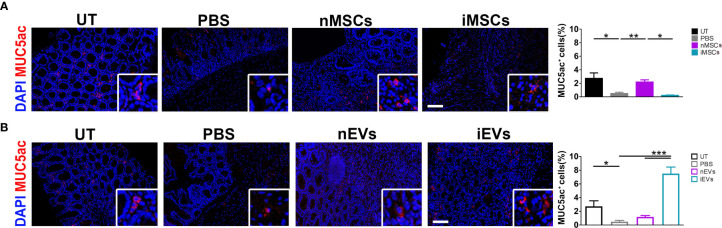
iEVs strongly enhance Mucin 5ac in mice with colitis. **(A)** Quantification of MUC5ac in colon mucosa in nMSCs and iMSCs mice: IF (left), quantification (right). **(B)** Quantification of mucin 5ac in colon mucosa in nEVs and iEVs mice: IF (left), quantification (right). Results are mean ± standard error (n = 5). *P < 0.05, **P < 0.01, ***P < 0.001.

In order to investigate the intestinal mucosa at functional level, the expression of mucin 5ac was analyzed. The treatment with DSS significantly reduced mucin 5ac expression, suggesting loss of functionality. However, this protein increased in colon mucosa of mice receiving unprimed MSCs in respect to PBS treated animals (**Figure 7A**), while iMSCs had no effect (**Figure 7A**). Of note, a strong 8-fold induction of MUC5ac was observed in mice receiving iEVs, suggesting a regenerative action of iEVs, yet the administration of nEVs had no significant effect (**Figure 7B**).

## Discussion

Chronic intestinal inflammation results from the interaction of genetic, immunological and environmental factors. Recently, Mesenchymal Stromal Cells (MSCs) have been approved by the European Medicines Agency for the treatment for Crohn’s fistulas resistant to conventional therapy and biologics. The effectiveness of MSC therapy probably relies on its complex immune regulatory rather than suppressive properties, being able of redirecting both the innate and the adaptive and innate immune system towards an anti-inflammatory, pro-regenerative response. However, cell dosages and timing are still based on insufficient experience and may be limited by high production costs (33). Even more critical, administered MSCs may behave differently in different patients. MSCs secrete soluble factors such as indoleamine 2,3-dioxygenase (IDO), involved in the catabolism of the essential amino acid tryptophan (34). This results in the degradation of tryptophan and accumulation of toxic kynurenines with inhibition of T cell proliferation. Upregulation of stress response pathways such as inducible nitric-oxide synthetase (iNOS) variably contributes to MSCs-induced immune suppression, with notable species differences (5). During MSCs-mediated immunomodulation, proinflammatory cytokines have been shown to play a key role, provoking MSCs to express iNOS (in rodents) or IDO (in humans) associated with T cell suppression. Waterman et al. (7) described a different immunomodulatory mechanism of MSCs that can polarize to a proinflammatory or to an immunosuppressive phenotype depending respectively on Toll-like receptor (TLR) 4 or TLR3 priming, thus again depending on the characteristics of the inflammatory environment. These findings suggest that it is important to be aware of the potential differential effects of cytokines or drugs on the expression and activity of IDO when applying MSCs in the treatment of disease, as they are a critical switch that determine the immunomodulatory fate of MSCs.

Several reports confirmed the immunomodulatory and pro-regenerative effects of MSCs and their secreted EVs. However, we obtained strikingly different results in our well-established murine model of DSS-induced colitis. In this experimental setup, administration of MSCs (both unprimed and cytokine-conditioned) worsened the clinical signs of colitis, as shown by a decreased body wt and by a trend to an increased DAI. Moreover, the histomorphometric scores of colitis were significantly worsened in both MSC-treated groups. It was reported that the therapeutic efficacy of MSCs in experimental colitis could be improved by priming with pro-inflammatory cytokines (35, 36). We have thus treated a group of mice with MSCs conditioned with a cytokine cocktail (iMSCs) known to enhance the cell immunomodulatory and anti-angiogenic activity (20, 21). However, iMSC administration also worsened colitis compared with the PBS-treated (control) group. Even if several reports describe the therapeutic efficacy of MSCs in DSS-induced colitis (29–31, 37–39) a worsening effect of these cells in the present model was reported by other investigators (40). It was also reported that MSCs can revert to a pro-inflammatory phenotype in collagen-induced arthritis (41, 42). It should be noticed that we used murine (syngeneic) MSCs in order to avoid possible bias due to species differences, as in the work of Nam et al. (40). However, improvement of DSS-induced colitis was described following administration of syngeneic (31, 43), allogeneic (37, 38) and xenogeneic (human) MSCs (29). The opposite effects observed by us and by Nam et al. are probably resulting from the poor reproducibility of the experimental model that can be influenced by strain differences and by environmental factors, the latter possibly affecting the composition of intestinal microbiota and thus the local immune response (44, 45). However, our results confirm that the effect of exogenous MSCs can be unpredictable, likely depending on poorly understood host environmental factors.

Administration of EVs secreted by unprimed nMSCs was not effective in ameliorating clinical and histomorphometric indexes of colitis, although a trend to improvement was present both in the DAI and in the histomorphometric score. However, administration of iEVs, obtained from cytokine-conditioned MSCs, was associated with a significant improvement in all clinicopathological parameters of disease.

The effects of cytokine priming on MSCs and the composition of iEVs vs. nEVs were characterized previously (23, 46). MSC stimulation with the cytokine cocktail described here resulted in the enhanced secretion of several specific proteins involved in inflammation and angiogenesis (46), including matrix metalloproteases (MMPs), which are considered both effectors and regulators of several biological processes since they can activate, inactivate or antagonize growth factors, cytokines and chemokines by proteolytic processing. In a more recent study, it was demonstrated that cytokine-stimulated MSCs produce EVs that are enriched in the anti-angiogenic metalloprotease TIMP-1 (23). Moreover, these iEVs carry CD39 and are particularly enriched in CD73 (23). Since the combination of CD39 and CD73 degrade ATP, ADP, and AMP to adenosine, these iEVs might be viewed as “immunological switches” that shift ATP-driven pro-inflammatory immune cell activity toward an anti-inflammatory state mediated by adenosine (47). Extracellular adenosine reduces the expression of adhesion molecules in endothelial cell, thus preventing the recruitment of leukocytes into the injured tissue (48, 49). Angioni et al. (23) also identified a novel anti-angiogenic mechanism of iEVs based on adenosine production, triggering of A2B adenosine receptors, and induction of NOX2-dependent oxidative stress (leading to apoptosis) within endothelial cells. In the present work we confirmed the anti-angiogenic effect of iEVs in an *in vitro* tube formation assay and demonstrated that these nanoparticles can also polarize macrophages to an anti-inflammatory phenotype *in vitro* (**Figure 1**). Thus, MSC stimulation with the present cytokine cocktail results in the production of EVs targeting both inflammation and angiogenesis at several levels.

Previous reports in animal models of experimental colitis (50, 51) described beneficial effects of allogeneic (rat) or xenogeneic (human to mice) bone marrow-derived MSC-EVs. We cannot exclude that the trend to improvement observed in our nEV treated group could become significant with higher EV dosages. MSC dose in mice was in the order of 10^6^, similar to the one shown to be effective in previous publications on the treatment of DSS-induced colitis (29–31). As described in the manuscript, in choosing the EV dose we empirically applied the cell/EV ratio of 1:1000. This EV dose per Kg body wt was shown to be effective in animal models of lung injury in previous publications by others and by us (32, 52, 53) and in a patient with GVHD (54). However, we clearly demonstrated that, at similar dosages, the therapeutic efficacy of EVs is greatly enhanced by cytokine preconditioning of the parent cells. More importantly, we showed that MSC-EVs exert a beneficial effect in the same pathologic environment that is on the contrary adversely affected by administration of their parent cells. To our knowledge, this is the first *in vivo* demonstration that EVs can produce opposite effects with respect to their cells of origin. Moreover, these findings suggest that the effects of EVs can be more reproducible than their parent cells. Indeed, unlike cells, EVs carry a definite set of signals, which should not be modified by exposure to host environmental factors.

Colitis induced by short-term DSS administration is an acute model of mucosal inflammation involving innate immune mechanisms, resulting in macrophage and neutrophil infiltration (45). Therefore, this model does not mirror the complexity of chronic IBD. However, the central role of macrophages in the pathogenesis of IBD is well recognized (39, 55). The expression of iNOS2, a molecular marker of M1 activation, was increased following induction of colitis and remained high in the two MSC- treated groups, consistently with the clinicopathological data. The expression of CD 163 as a marker of M2 activation was more variable, showing an increase in the nMSC-treated group and a decrease in the iMSC-treated mice. An opposite behavior was again observed in the EV-treated groups: the M1 activation marker was significantly decreased in both nEV-treated and iEV-treated mice, and the M2 activation marker was significantly increased in the iEV-treated mice. Clearly, the complexity of the macrophage activation states cannot be fully described by changes in the above molecular markers. However, such changes are consistent with the clinicopathological improvements observed in EV-treated mice and with lymphocyte profile in the intestinal lymph node. As discussed above, nMSCs tended to polarize the macrophages to an anti-inflammatory phenotype. However, only EVs suppressed the expression of iNOS, a major effector of inflammation. This observation might help explaining the differential effects of MSCs and of their secreted EVs on the Treg/Teff ratio and on clinical and histological findings. Clearly, the mechanisms underlying the effect of EVs on iNOS are currently unknown and require further investigation. The development of colitis was associated with a reduced Treg/Teff ratio, a finding that was not modified in the groups treated with MSCs. The reduction of the Treg/Teff ratio was also unaffected by nEV administration, but was reversed in iEV-treated mice, consistently with the increased M2 macrophage polarization observed in this group.

Inflammation, angiogenesis and collagen deposition that, with time, strengthen fibrogenesis, are intimately intertwined processes. Dysregulated angiogenesis is a hallmark of pathological processes such as chronic inflammation and cancer (56, 57). Our results with high expression of CD31 in nEVs treated mice and low expression of the angiogenic protein in iEVs animals, suggest that the increased therapeutic efficacy of iEVs compared to nEVs in the present experimental model is probably multifactorial. The apparent inconsistency between CD31% area and RQ expression (**Figure 6**) could be explained with a diminished gene expression in this time window, i.e. 3 days after interrupting DSS administration, while the translated protein is still present in the tissue, We have recently observed that EVs secreted by MSCs primed with IL1β, IL6 and TNFα exhibit increased expression of CD73 (23). CD73 acts in concert with CD39 to degrade ATP, ADP, and AMP to adenosine, shifting the ATP-driven pro-inflammatory immune response toward an anti-inflammatory condition mediated by adenosine (47). Therefore, amelioration of colitis could be at least partially mediated by such enhanced anti-inflammatory activity of iEVs. However, we also observed that iEVs exert a strong anti-angiogenic activity mediated by the expression of TIMP-1 peptide (23), consistently with the concept that inflammation and angiogenesis are intimately interlinked (57, 58). In particular, angiogenesis is an important component in the pathogenesis of IBD (59) and anti-angiogenic therapies have been used in this disorder (60). Moreover, it was shown that treatment with anti-angiogenic peptides ameliorates DSS-induced colitis (61, 62). The anti-angiogenic activity of iEVs could thus represent an additional mechanism responsible for the therapeutic effects observed in the present work.

Tissue fibrosis, with connective tissue replacing normal parenchymal tissue, can result from excessive or dysregulated inflammation (63). Scar formation resulting in intestinal obstruction is a major complication In Crohn’s disease (64). Collagen deposition was increased following induction of colitis and was further increased in MSC-treated mice. On the contrary, iEV-treated animals showed diminished collagen, likely resulting from the reduced inflammatory injury observed in this group.

The mucus layer lining the epithelial surface of the intestine has several functions, including the control of the passage of nutrients and the regulation of host interactions with the intestinal microbiota (65, 66). Secreted mucins are the major proteins found in the mucus layer, with MUC2 being largely predominant in the healthy intestinal mucosa, while MUC5AC (human)/Muc5ac (murine) is alternatively secreted in IBD and in experimental colitis (67). Previous studies both in mice and in patients with IBD suggest that Muc5ac/MUC5AC expression in the gastrointestinal tract is a tissue-protective response during active inflammation (67, 68). Here, we show that MUC5ac was reduced following the induction of chemical colitis, probably because of mucosal cell destruction and of the detergent effect of DSS on the mucus layer. Interestingly, administration of iEVs resulted in a striking 8-fold increase of Muc5ac, likely because of reduced epithelial injury and increased mucus secretion. Increased mucus secretion could help restoring the intestinal barrier, thus contributing to the observed reduction in mucosal inflammation. Regulation of MUC5ac secretion is poorly understood (67). Therefore, the mechanisms underlying these novel findings are presently unknown and require further investigation. Of note, nMSC administration was also associated with a much lower but significant increase in MUC5ac production. It thus seems that nMSCs do exert some anti-inflammatory and pro-regenerative effects, but such effects seem insufficient to overcome the strong inflammatory condition associated with this animal model, as reported by others (40).

We should however emphasize that the present work compares very different products. Since the composition of secreted EVs is dependent on the response of the cells to the environment (69), the EVs produced by MSCs *in vitro* are likely quite different from those produced by the same cells *in vivo*. Moreover, it is known that most exogenous cells will die within hours from transplantation (70–72), further affecting both the quality and the quantity of secreted EVs.

A major limitation of this study is the lack of a dose-response assessment. In particular, administration of higher EV doses is required to define the therapeutic range and to explore possible side effects.

In summary, we show for the first time that EVs derived from cytokine-primed MSCs can exert beneficial effects *in vivo* in the same inflammatory condition that is on the contrary adversely affected by their cells of origin. Our findings also suggest that the effects of nEVs could be more reproducible with respect to their cells of origin, supporting the concept of a better safety profile in favor of these nanoparticles (73). In the present model, EVs confirmed their pleiotropic effects, affecting several different immunological pathways. However, additional mechanistic studies are needed to dissect the complex mode of action of these nanoparticles in inflammatory conditions.

## Data Availability Statement

The original contributions presented in the study are included in the article/**Supplementary Material**. Further inquiries can be directed to the corresponding authors.

## Ethics Statement

The animal study was reviewed and approved by Italian Ministry of Health, Division of Veterinary Medicine (protocol n°861/2016-pr, risp. a prot c35de.2 #195042387#).

## Author Contributions

AMT, MPo, MM: conception and design of the experiments, collection and assembling of the data, data analysis and interpretation, manuscript writing. IC, MPi, MG, CF. conception and design of the experiments, collection and assembling of the data. FM, GDL, RM, FC, MS: collection and assembling of the data. VM, RDC, IA, AV, AP: analysis and interpretation of the data. All authors contributed to the article and approved the submitted version.

## Funding

This work was supported by the Crohn’s and Colitis Foundation of America (grant no. 383514) AT is supported by the ‘Consorzio per la Ricerca Sanitaria’ (CORIS) of the Veneto Region, Italy (L.i.f.e.L.a.b. Program), grant number DGR1017, July 17, 2018. FM is recipient of a fellowship from the Institute of Pediatric Research “Città della Speranza” (Synergy Grant 19/01). RM is recipient of the fellowship 40/1939 from the “Istituto Italo-Latino Americano”. MP is supported by University of Padova, Tenure track L.n. 240/10.

## Conflict of Interest

The authors declare that the research was conducted in the absence of any commercial or financial relationships that could be construed as a potential conflict of interest.

## References

[1] Thoreson R Cullen JJ. Pathophysiology of Inflammatory Bowel Disease: An Overview. Surg Clin North Am (2007) 87:575–85. 10.1016/j.suc.2007.03.001 17560413

[2] Kaistha A Levine J. Inflammatory Bowel Disease: The Classic Gastrointestinal Autoimmune Disease. Curr Probl Pediatr Adolesc Health Care (2014) 44:328–34. 10.1016/j.cppeds.2014.10.003 25499459

[3] Chudy-Onwugaje KO Christian KE Farraye FA Cross RK. A state-of-the-art review of new and emerging therapies for the treatment of IBD. Inflamm Bowel Dis (2019) 25:820–30. 10.1093/ibd/izy327 PMC6468492 30445504

[4] Panés J García-Olmo D Van Assche G Colombel JF Reinisch W Baumgart DC. Expanded allogeneic adipose-derived mesenchymal stem cells (Cx601) for complex perianal fistulas in Crohn’s disease: a phase 3 randomised, double-blind controlled trial. Lancet (2016) 388:1281–90. 10.1016/S0140-6736(16)31203-X 27477896

[5] Ren G Su J Zhang L Zhao X Ling W L’Huillie A. Species variation in the mechanisms of mesenchymal stem cell-mediated immunosuppression. Stem Cells (2009) 27:1954–62. 10.1002/stem.118 19544427

[6] Li W Ren G Huang Y Su J Han Y Li J. Mesenchymal stem cells: A double-edged sword in regulating immune responses. Cell Death Differ (2012) 19:1505–13. 10.1038/cdd.2012.26 PMC3422473 22421969

[7] Waterman RS Tomchuck SL Henkle SL Betancourt AM. A New Mesenchymal Stem Cell (MSC) Paradigm: Polarization into a Pro-Inflammatory MSC1 or an Immunosuppressive MSC2 Phenotype. PloS One (2010) 5:e10088. 10.1371/journal.pone.0010088 20436665 PMC2859930

[8] Doorn J Moll G Le Blanc K Van Blitterswijk C De Boer J. Therapeutic applications of mesenchymal stromal cells: Paracrine effects and potential improvements. Tissue Eng Part B Rev (2012) 18:101–15. 10.1089/ten.teb.2011.0488 21995703

[9] Mirotsou M Jayawardena TM Schmeckpeper J Gnecchi M Dzau VJ. Paracrine mechanisms of stem cell reparative and regenerative actions in the heart. J Mol Cell Cardiol (2011) 50:280–9. 10.1016/j.yjmcc.2010.08.005 PMC3021634 20727900

[10] Liang X Ding Y Zhang Y Tse HF Lian Q. Paracrine mechanisms of mesenchymal stem cell-based therapy: Current status and perspectives. Cell Transplant (2014) 23:1045–59. 10.3727/096368913X667709 23676629

[11] György B Szabó TG Pásztói M Pál Z Misják P Aradi B. Membrane vesicles, current state-of-the-art: Emerging role of extracellular vesicles. Cell Mol Life Sci (2011) 68:2667–88. 10.1007/s00018-011-0689-3 PMC3142546 21560073

[12] Colombo M Raposo G Théry C. Biogenesis, Secretion, and Intercellular Interactions of Exosomes and Other Extracellular Vesicles. Annu Rev Cell Dev Biol (2014) 30:255–89. 10.1146/annurev-cellbio-101512-122326 25288114

[13] Chaput N Théry C. Exosomes: Immune properties and potential clinical implementations. Semin Immunopathol (2011) 33:419–40. 10.1007/s00281-010-0233-9 21174094

[14] Théry C Witwer KW Aikawa E Alcaraz MJ Anderson JD Andriantsitohaina R. Minimal information for studies of extracellular vesicles 2018 (MISEV2018): a position statement of the International Society for Extracellular Vesicles and update of the MISEV2014 guidelines. J Extracell Vesicles (2018) 7:7–43. 10.1080/20013078.2018.1535750 PMC6322352 30637094

[15] Phinney DG Pittenger MF. Concise Review: MSC-Derived Exosomes for Cell-Free Therapy. Stem Cells (2017) 35:851–8. 10.1002/stem.2575 28294454

[16] Renner P Eggenhofer E Rosenauer A Popp FC Steinmann JF Slowik P. Mesenchymal Stem Cells Require a Sufficient, Ongoing Immune Response to Exert Their Immunosuppressive Function. Transplant Proc (2009) 41:2607–11. 10.1016/j.transproceed.2009.06.119 19715984

[17] Sivanathan KN Rojas-Canales DM Hope CM Krishnan R Carroll RP Gronthos S. Interleukin-17A-Induced Human Mesenchymal Stem Cells Are Superior Modulators of Immunological Function. Stem Cells (2015) 33:2850–63. 10.1002/stem.2075 26037953

[18] Prasanna SJ Gopalakrishnan D Shankar SR Vasandan AB. Pro-Inflammatory Cytokines, IFNγ and TNFα, Influence Immune Properties of Human Bone Marrow and Wharton Jelly Mesenchymal Stem Cells Differentially. PloS One (2010) 5:e9016. 10.1371/journal.pone.0009016 20126406 PMC2814860

[19] de Witte SFH Merino AM Franquesa M Strini T van Zoggel JAA Korevaar SS. Cytokine treatment optimises the immunotherapeutic effects of umbilical cord-derived MSC for treatment of inflammatory liver disease. Stem Cell Res Ther (2017) 8:1–12. 10.1186/S13287-017-0590-6 28595619 PMC5465593

[20] Zanotti L Angioni R Calì B Soldani C Ploia C Moalli F. Mouse mesenchymal stem cells inhibit high endothelial cell activation and lymphocyte homing to lymph nodes by releasing TIMP-1. Leukemia (2016) 30:1143–54. 10.1038/leu.2016.33 PMC4858586 26898191

[21] Di Trapani M Bassi G Midolo M Gatti A Kamga PT Cassaro A. Differential and transferable modulatory effects of mesenchymal stromal cell-derived extracellular vesicles on T, B and NK cell functions. Sci Rep (2016) 6:1–13. 10.1038/srep24120 27071676 PMC4829861

[22] Kilpinen L Impola U Sankkila L Ritamo I Aatonen M Kilpinen S. Extracellular membrane vesicles from umbilical cord blood-derived MSC protect against ischemic acute kidney injury, a feature that is lost after inflammatory conditioning. J Extracell Vesicles (2013) 2:21927. 10.3402/jev.v2i0.21927 PMC3860334 24349659

[23] Angioni R Liboni C Herkenne S Sánchez-Rodríguez R Borile G Marcuzzi E. CD73+ extracellular vesicles inhibit angiogenesis through adenosine A2B receptor signalling. J Extracell Vesicles (2020) 9:1757900. 10.1080/20013078.2020.1757900 32489531 PMC7241475

[24] Chen Y Li C Tan C Liu X. Circular RNAs: A new frontier in the study of human diseases. J Med Genet (2016) 53:359–65. 10.1136/jmedgenet-2016-103758 26945092

[25] Eggenhofer E Benseler V Kroemer A Popp FC Geissler EK Schlitt HJ. Mesenchymal stem cells are short-lived and do not migrate beyond the lungs after intravenous infusion. Front Immunol (2012) 3:297. 10.3389/fimmu.2012.00297 23056000 PMC3458305

[26] Murthy SN Shah RS Sedergran DJ CH. Clinicopathologic study of dextran sulfate sodium experimental murine colitis - PubMed. Available at: https://pubmed.ncbi.nlm.nih.gov/8350599/ (Accessed November 2, 2020). 8350599

[27] Dominici M Le Blanc K Mueller I Slaper-Cortenbach I Marini FC Krause DS. Minimal criteria for defining multipotent mesenchymal stromal cells. The International Society for Cellular Therapy position statement. Cytotherapy (2006) 8:315–7. 10.1080/14653240600855905 16923606

[28] Osteikoetxea X Sódar B Németh A Szabó-Taylor K Pálóczi K Vukman KV. Differential detergent sensitivity of extracellular vesicle subpopulations. Org Biomol Chem (2015) 13:9775–82. 10.1039/c5ob01451d 26264754

[29] González MA Gonzalez-Rey E Rico L Büscher D Delgado M. Adipose-Derived Mesenchymal Stem Cells Alleviate Experimental Colitis by Inhibiting Inflammatory and Autoimmune Responses. Gastroenterology (2009) 136:978–89. 10.1053/j.gastro.2008.11.041 19135996

[30] Gonçalves F da C. Intravenous *vs* intraperitoneal mesenchymal stem cells administration: What is the best route for treating experimental colitis? World J Gastroenterol (2014) 20:18228. 10.3748/wjg.v20.i48.18228 25561790 PMC4277960

[31] He XW He XS Lian L Wu XJ Lan P. Systemic infusion of bone marrow-derived mesenchymal stem cells for treatment of experimental colitis in mice. Dig Dis Sci (2012) 57:3136–44. 10.1007/s10620-012-2290-5 22752635

[32] Porzionato A Zaramella P Dedja A Guidolin D Van Wemmel K Macchi V. Intratracheal administration of clinical-grade mesenchymal stem cell-derived extracellular vesicles reduces lung injury in a rat model of bronchopulmonary dysplasia. Am J Physiol Lung Cell Mol Physiol (2019) 316:L6–L19. 10.1152/ajplung.00109.2018 30284924

[33] Alofisel may offer a much needed treatment for fistulizing CD patients, but will it be cost-effective? | DRG Blog, Drug Watch | DRG. Available at: https://decisionresourcesgroup.com/blog/alofisel-much-needed-cost-effective-fistulizing-crohns-disease/ (Accessed November 7, 2020).

[34] Meisel R Zibert A Laryea M Göbel U Däubener W Dilloo D. Human bone marrow stromal cells inhibit allogeneic T-cell responses by indoleamine 2,3-dioxygenase-mediated tryptophan degradation. Blood (2004) 103:4619–21. 10.1182/blood-2003-11-3909 15001472

[35] Fan H Zhao G Liu L Liu F Gong W Liu X. Pre-treatment with IL-1β enhances the efficacy of MSC transplantation in DSS-induced colitis. Cell Mol Immunol (2012) 9:473–81. 10.1038/cmi.2012.40 PMC4002219 23085948

[36] Chen H Min XH Wang QY Leung FW Shi L Zhou Y. Pre-activation of mesenchymal stem cells with TNF-α, IL-1β 2 and nitric oxide enhances its paracrine effects on radiation-induced intestinal injury. Sci Rep (2015) 5:1–14. 10.1038/srep08718 PMC4346809 25732721

[37] Tanaka F Tominaga K Ochi M Tanigawa T Watanabe T Fujiwara Y. Exogenous administration of mesenchymal stem cells ameliorates dextran sulfate sodium-induced colitis via anti-inflammatory action in damaged tissue in rats. Life Sci (2008) 83:771–9. 10.1016/j.lfs.2008.09.016 18950645

[38] Castelo-Branco MTL Soares IDP Lopes DV Buongusto F Martinusso CA do Rosario A. Intraperitoneal but Not Intravenous Cryopreserved Mesenchymal Stromal Cells Home to the Inflamed Colon and Ameliorate Experimental Colitis. PloS One (2012) 7:e33360. 10.1371/journal.pone.0033360 22432015 PMC3303821

[39] Wang C Chen J Sun L Liu Y. TGF-beta signaling-dependent alleviation of dextran sulfate sodium-induced colitis by mesenchymal stem cell transplantation. Mol Biol Rep (2014) 41:4977–83. 10.1007/s11033-014-3364-6 24737572

[40] Nam Y-S Kim N Im K-I Lim J-Y Lee E-S Cho S-G. Negative impact of bone-marrow-derived mesenchymal stem cells on dextran sulfate sodium-induced colitis. World J Gastroenterol (2015) 21:2030–9. 10.3748/wjg.v21.i7.2030 PMC4326137 25717235

[41] Djouad F Fritz V Apparailly F Louis-Plence P Bony C Sany J. Reversal of the immunosuppressive properties of mesenchymal stem cells by tumor necrosis factor α in collagen-induced arthritis. Arthritis Rheum (2005) 52:1595–603. 10.1002/art.21012 15880818

[42] Duijvestein M Molendijk I Roelofs H Vos ACW Verhaar AP Reinders MEJ. Mesenchymal stromal cell function is not affected by drugs used in the treatment of inflammatory bowel disease. Cytotherapy (2011) 13:1066–73. 10.3109/14653249.2011.597379 21846292

[43] Silva AM Teixeira JH Almeida MI Gonçalves RM Barbosa MA Santos SG. Extracellular Vesicles: Immunomodulatory messengers in the context of tissue repair/regeneration. Eur J Pharm Sci (2017) 98:86–95. 10.1016/j.ejps.2016.09.017 27644894

[44] Chassaing B Aitken JD Malleshappa M Vijay-Kumar M. Dextran sulfate sodium (DSS)-induced colitis in mice. Curr Protoc Immunol (2014) 104:15.25.1–15.25.14. 10.1002/0471142735.im1525s104 24510619 PMC3980572

[45] Kiesler P Fuss IJ Strober W. Experimental models of inflammatory bowel diseases. Med Hyg (Geneve) (2001) 59:241–8. 10.1016/j.jcmgh.2015.01.006 PMC4435576 26000334

[46] Maffioli E Nonnis S Angioni R Santagata F Calì B Zanotti L. Proteomic analysis of the secretome of human bone marrow-derived mesenchymal stem cells primed by pro-inflammatory cytokines. J Proteomics (2017) 166:115–26. 10.1016/j.jprot.2017.07.012 28739509

[47] Antonioli L Pacher P Vizi ES Haskó G. CD39 and CD73 in immunity and inflammation. Trends Mol Med (2013) 19:355–67. 10.1016/j.molmed.2013.03.005 PMC3674206 23601906

[48] Bouma MG Van Den Wildenberg FAJM Buurman WA. Adenosine inhibits cytokine release and expression of adhesion molecules by activated human endothelial cells. Am J Physiol Cell Physiol (1996) 270(2pt):C522–9. 10.1152/ajpcell.1996.270.2.c522 8779915

[49] Bours MJL Swennen ELR Di Virgilio F Cronstein BN Dagnelie PC. Adenosine 5′-triphosphate and adenosine as endogenous signaling molecules in immunity and inflammation. Pharmacol Ther (2006) 112:358–404. 10.1016/j.pharmthera.2005.04.013 16784779

[50] Yang J Liu X-X Fan H Tang Q Shou Z-X Zuo D-M. Extracellular Vesicles Derived from Bone Marrow Mesenchymal Stem Cells Protect against Experimental Colitis via Attenuating Colon Inflammation, Oxidative Stress and Apoptosis. PloS One (2015) 10:e0140551. 10.1371/journal.pone.0140551 26469068 PMC4607447

[51] Mao F Wu Y Tang X Kang J Zhang B Yan Y. Exosomes Derived from Human Umbilical Cord Mesenchymal Stem Cells Relieve Inflammatory Bowel Disease in Mice. BioMed Res Int (2017) 2017:1–12. 10.1155/2017/5356760 PMC5447283 28589143

[52] Porzionato A Zaramella P Dedja A Guidolin D Bonadies L Macchi V. Intratracheal administration of mesenchymal stem cell-derived extracellular vesicles reduces lung injuries in a chronic rat model of bronchopulmonary dysplasia. Am J Physiol Cell Mol Physiol (2021). 10.1152/ajplung.00148.2020 33502939

[53] Willis GR Fernandez-Gonzalez A Anastas J Vitali SH Liu X Ericsson M. Mesenchymal stromal cell exosomes ameliorate experimental bronchopulmonary dysplasia and restore lung function through macrophage immunomodulation. Am J Respir Crit Care Med (2018) 197:104–16. 10.1164/rccm.201705-0925OC PMC5765387 28853608

[54] Kordelas L Rebmann V Ludwig AK Radtke S Ruesing J Doeppner TR. MSC-derived exosomes: A novel tool to treat therapy-refractory graft-versus-host disease. Leukemia (2014) 28:970–3. 10.1038/leu.2014.41 24445866

[55] Bain CC Mowat AMI. The monocyte-macrophage axis in the intestine. Cell Immunol (2014) 291:41–8. 10.1016/j.cellimm.2014.03.012 PMC4217150 24726741

[56] Szade A Grochot-Przeczek A Florczyk U Jozkowicz A Dulak J. Cellular and molecular mechanisms of inflammation-induced angiogenesis. IUBMB Life (2015) 67:145–59. 10.1002/iub.1358 25899846

[57] Aguilar-Cazares D Chavez-Dominguez R Carlos-Reyes A Lopez-Camarillo C Hernadez de la Cruz ON Lopez-Gonzalez JS. Contribution of Angiogenesis to Inflammation and Cancer. Front Oncol (2019) 9:1399. 10.3389/fonc.2019.01399 31921656 PMC6920210

[58] Szewczyk G Rak J Ruth JH. Inflammatory mediators of angiogenesis. Mediators Inflamm (2013) 2013:1–2. 10.1155/2013/610543 PMC3791823 24163506

[59] Alkim C Alkim H Koksal AR Boga S Sen I. Angiogenesis in inflammatory bowel disease. Int J Inflam (2015) 2015:1–10. 10.1155/2015/970890 PMC4709626 26839731

[60] Chidlow JH Shukla D Grisham MB Kevil CG. Pathogenic angiogenesis in IBD and experimental colitis: New ideas and therapeutic avenues. Am J Physiol Gastrointest Liver Physiol (2007) 293:G5–G18. 10.1152/ajpgi.00107.2007 17463183

[61] Gutierrez LS. Thrombospondin peptide ABT-898 inhibits inflammation and angiogenesis in a colitis model. World J Gastroenterol (2015) 21:6157. 10.3748/wjg.v21.i20.6157 26034351 PMC4445093

[62] Punekar S Zak S Kalter VG Dobransky L Punekar I Lawler JW. Thrombospondin 1 and Its Mimetic Peptide ABT-510 Decrease Angiogenesis and Inflammation in a Murine Model of Inflammatory Bowel Disease. Pathobiology (2008) 75:9–21. 10.1159/000113790 18334835

[63] Weiskirchen R Weiskirchen S Tacke F. Organ and tissue fibrosis: Molecular signals, cellular mechanisms and translational implications. Mol Aspects Med (2019) 65:2–15. 10.1016/j.mam.2018.06.003 29958900

[64] Gasche C. Complications of inflammatory bowel disease. Hepatogastroenterology (2000) 47:49–56. 10.1093/med/9780199231362.003.0007 10690585

[65] Sánchez de Medina F Romero-Calvo I Mascaraque C Martínez-Augustin O. Intestinal Inflammation and Mucosal Barrier Function. Inflamm Bowel Dis (2014) 20:2394–404. 10.1097/MIB.0000000000000204 25222662

[66] Johansson MEV Hansson GC. Immunological aspects of intestinal mucus and mucins. Nat Rev Immunol (2016) 16:639–49. 10.1038/nri.2016.88 PMC6435297 27498766

[67] Olli KE Rapp C O’Connell L Collins CB McNamee EN Jensen O. Muc5ac Expression Protects the Colonic Barrier in Experimental Colitis. Inflamm Bowel Dis (2020) 26:1353–67. 10.1093/ibd/izaa064 PMC7441107 32385500

[68] Hasnain SZ Evans CM Roy M Gallagher AL Kindrachuk KN Barron L. Muc5ac: A critical component mediating the rejection of enteric nematodes. J Exp Med (2011) 208:893–900. 10.1084/jem.20102057 21502330 PMC3092342

[69] Van Niel G D’Angelo G Raposo G. Shedding light on the cell biology of extracellular vesicles. Nat Rev Mol Cell Biol (2018) 19:213–28. 10.1038/nrm.2017.125 29339798

[70] Li L Chen X Wang WE Zeng C. How to Improve the Survival of Transplanted Mesenchymal Stem Cell in Ischemic Heart? Stem Cells Int (2016) 2016:1–14. 10.1155/2016/9682757 PMC4670674 26681958

[71] Toma C Pittenger MF Cahill KS Byrne BJ Kessler PD. Human mesenchymal stem cells differentiate to a cardiomyocyte phenotype in the adult murine heart. Circulation (2002) 105:93–8. 10.1161/hc0102.101442 11772882

[72] McGinley LM McMahon J Stocca A Duffy A Flynn A O’Toole D. Mesenchymal stem cell survival in the infarcted heart is enhanced by lentivirus vector-mediated heat shock protein 27 expression. Hum Gene Ther (2013) 24:840–51. 10.1089/hum.2011.009 PMC3787467 23987185

[73] Zipkin M. Exosome redux. Nat Biotechnol (2019) 37:1395–400. 10.1038/s41587-019-0326-5 31796920

