# Supplementary material for: Extracellular Vesicles Secreted by Mesenchymal Stromal Cells Exert Opposite Effects to Their Cells of Origin in Murine Sodium Dextran Sulfate-Induced Colitis
Source: Front Immunol. 2021 Apr 13;12:627605. doi: 10.3389/fimmu.2021.627605 (PMC8076641; doi:10.3389/fimmu.2021.627605)
Supplement: Supplementary file 1 [file DataSheet_1.pdf]

Supplementary Material

Supplementary Figure 1.

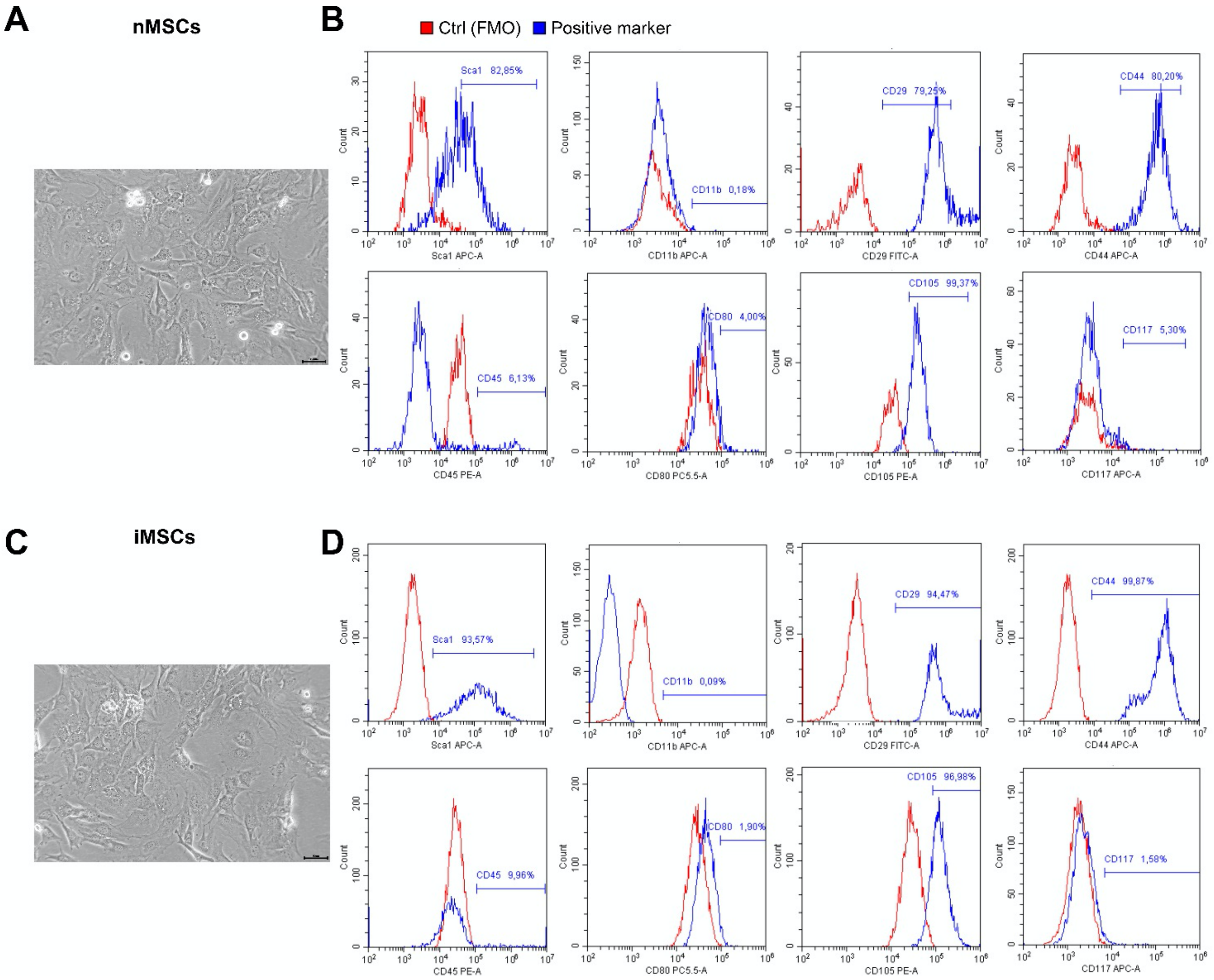

Supplementary Figure 1. (A) Phase contrast of nMSCs. Scale bar: 100µm. **(B)** Cytofluorimetric characterization of nMSC. The typical mesenchymal markers are highly expressed (SCA-1, CD105, CD29, CD44), as well as the hematopoietic markers are negative (CD11b, CD45, CD117, CD80). **(C)** Phase contrast of iMSCs. Scale bar: 100µm. **(D)** Cytofluorimetric characterization of iMSCs. The typical mesenchymal markers are highly expressed (SCA-1, CD105, CD29, CD44), as well as the

hematopoietic markers are negative (CD11b, CD45, CD117, CD80). FMO= Fluorescence Minus One, control.
